# Prevalence of BoHV-1 seropositive and BVD virus positive bulls on Irish dairy farms and associations between bull purchase and herd status

**DOI:** 10.1186/s13620-015-0059-9

**Published:** 2015-12-09

**Authors:** A. M. Martinez-Ibeas, Clare Power, Jennifer McClure, Riona G. Sayers

**Affiliations:** Animal and Biosciences Department, Animal & Grassland Research and Innovation Centre, Teagasc, Moorepark, Fermoy, Co. Cork, Ireland; Institute of Technology Carlow, Kilkenny Road, Carlow, Co. Carlow, Ireland; Irish Cattle Breeding Federation, Bandon, Co. Cork, Ireland

**Keywords:** Prevalence, BVD, BoHV-1, Bulls

## Abstract

**Background:**

BVD and IBR are contagious viral diseases highly prevalent in Irish cattle. Despite their significant reproductive and economic impact very little is known about the BVD and IBR status of stock bulls (a bull used for breeding purposes). There are still a high proportion of dairy farms in Ireland that rely on the use of a bull for breeding cattle and ensuring the fertility of the bulls is of paramount importance for the efficiency of the farms. The prevalence of BoHV-1 and BVD in stock bulls in Irish dairy herds has never been investigated. The objectives of this study therefore were: (i) to provide descriptive, observational data on the use of stock bulls on Irish dairy farms; (ii) to investigate the BVD and BoHV1 status of a sub-set of stock bulls; (iii) to investigate factors associated with BVD and BoHV1 status of stock bulls and (iv) to investigate factors associated with dairy herd status for BVD and BoHV1, including any associations with the use of stock bull.

A total of 529 blood samples from bulls involved in the dairy breeding process were analysed for BVD virus using RT-PCR, and BoHV-1 antibodies by ELISA test. A total of 305 different dairy herds took part in the study and the overall BVD and BoHV-1 herd status was determined by ELISA using four bulk tank milk samples over the 2009 lactation. Logistic regression was used to investigate the associations between the stock bulls and BVD and BoHV-1 herd and individual status.

**Results:**

Of the 305 total participating farms, 235 farms (77 %) had at least one bull and 167 farms had purchased bulls. Two bulls (0.4 %) out of 529 tested were found positive for BVD virus and 87 (16.7 %) tested seropositive for BoHV-1. Some significant associations were identified between the purchase of bulls and both viral diseases. Purchased bulls were three times more likely to be seropositive for BoHV-1 than homebred bulls. In the same way, herds with purchased bulls were three times more likely to be classified as seropositive for BVD and four times more likely to have evidence of recent BoHV-1 circulation than farms where all the bulls were homebred.

**Conclusions:**

The prevalence of BoHV-1 and BVD in stock bulls in Irish dairy herds has never been investigated. This study highlights the widespread use of stock bulls in Irish dairy herds, as well as the high rate of exchange of bulls between farms. Significant associations were found between the origin of the bull and their serological BoHV-1 status. In keeping with these results, bulls with higher number movements between farms were more likely to be seropositive for BoHV-1.

## Background

Artificial insemination (AI) has become the breeding norm on the majority of dairy farms in the Republic of Ireland [1]. However, the stock bull (a bull used for breeding purposes) also remains an integral part of the breeding process on many farms, either being relied on as a sole means of impregnating cows or as an adjunct to AI later in the breeding season [2]. Milk production systems in Ireland are primarily pasture-based and involve seasonal calving [3]. Over 80 % of Irish dairy farms are spring-calving with the majority of cows calving down in the first 6 months of the year and 21 % of all calves are born in March alone [4]. Compact spring-calving is essential to the efficiency of farms which operate extensive grass-based systems as calving is timed to coincide with the period of maximal grass growth [3]. Calves born later in the year will reduce farm efficiency [5].

A key breeding goal in a spring-calving system is to achieve high 3 and 6-week pregnancy rates from breeding start date to achieve a concentrated calving pattern the next season [6]. Bulls can be an efficient means of getting cows in calf as the breeding season progresses and also reduce the need for continuing heat detection [2]. Use of 6 weeks of AI followed by release of bulls to complete the breeding season is, therefore, a common reproductive strategy on many Irish dairy farms [1]. For the purposes of genetic diversity, these bulls are often purchased and are only introduced to the female breeding herd during the breeding season. Ensuring the health of these bulls is of paramount importance, therefore, in terms of bull fertility and disease transmission between cows and bulls. Bovine Viral Diarrhoea (BVD) and Infectious Bovine Rhinotracheitis (IBR) caused by BVD virus (BVDv) and bovine herpesvirus 1 (BoHV-1), respectively, are highly contagious diseases of economic and trade importance. Both are listed as notifiable diseases by the World Organisation for Animal Health (OIE) and are prevalent in Ireland [7]. Several European countries have implemented programmes to eradicate both diseases to facilitate free trade of cattle, semen, and embryos within the European Union (EU). Ireland embarked on a compulsory national eradication programme for BVD in 2013 based on identification of BVD persistently infected (PI) calves. Movement restrictions are placed on individuals testing BVD virus positive [8]. As of yet, no regulation exists for control and eradication of BoHV-1.

Cattle movement has been explored by many authors as a root cause of disease spread [9, 10]. Many Irish farmers operate what they perceive as closed herds i.e. no cattle introductions to the farm [7]. It has previously been highlighted, however, that discrepancies exist between what Irish dairy farmers perceive as a closed herd and a herd that does not engage in purchase of any cattle [7]. Bulls are often an ‘overlooked’ purchase in the maintenance of a closed herd. Breeding bulls, as with any purchased animal [9], are capable of introducing disease to a farm and may act as a vehicle through which diseases such as BVD and BoHV-1 can be transmitted. BVD and BoHV-1 can both adversely affect the fertility on a dairy farm with potentially devastating consequences for achieving a compact breeding season [11–14].

The levels of BVD and BoHV-1 infection amongst dairy stock bulls in Ireland have never been examined nor has the degree of bull movement between farms. Therefore, the objectives of this study were (i) to provide descriptive, observational data on the use of stock bulls on Irish dairy farms; (ii) to investigate the BVD and BoHV1 status of a sub-set of stock bulls; (iii) to investigate factors associated with BVD and BoHV1 status of stock bulls and (iv) to investigate factors associated with dairy herd status for BVD and BoHV1, including any associations with the use of stock bulls.

## Methods

### Study farm selection

The study was carried out in 2009 and licensed by the Irish Department of Health and Children meeting all legislative requirements for research involving animals in the Republic of Ireland at the time of the study.

A detailed description of the sample population used in this study has previously been outlined [15, 16]. Briefly, 500 herds from the Irish Cattle Breeding Federations (ICBF) database were invited to participate. Farms were randomly selected on the basis of a two-tier stratification protocol based on geographical location and herd size. All farms selected for inclusion were dairy farms and were registered on the ICBF’s database (a database of over 3,500 Irish dairy herds). Geographical regions were chosen based on the Irish Central Statistics Office (CSO) standard regions. Results of the national farm census conducted in 2000 were used to select farm size categories^1^.

### Sample collection and preparation

Study farms were visited to sample all bulls involved in the dairy breeding process. Bulls being raised for beef production were not included. Samples were taken by coccygeal venepuncture into plain glass vacutainers. Each sample was centrifuged at 5000 g for 5 min, serum aspirated and samples stored at −20 °C until analysed. Subsequently, a 100 μl aliquot of each serum sample was pooled in batches of ten for BVD virus testing. In addition, four bulk milk samples, and blood samples from 20 % of the replacement heifer group with a minimum of five heifers less than 9 months were sampled from each herd over the 2009 lactation These were used to determine herd BVD and BoHV-1 serostatus, both historical (bulk milk) and recent seroconversion (weanlings) [16].

### Sample testing

Pooled serum samples were tested for the presence of BVD virus by commercial laboratory (Enfer Labs Ltd, Ireland) using a Real Time–Polymerase Chain Reaction (RT-PCR). The detection limit of this assay was 50 to 100 viral particles in 1 ml whole blood [17]. Where a pooled serum batch yielded a PCR positive result, individual samples were re-tested, again by PCR, to identify the positive sample within the batch. Positive samples were tested individually to confirm the virus-positive status of each animal. Sera were tested for BoHV-1 antibody using the appropriate ELISA method depending on individual vaccination status (for bulls status) and herd vaccination status (for herd status) i.e. IBRgB (Ultrapurified BoHV-1 lysate, Institut Pourquier, France) in BoHV-1 unvaccinated bulls/herds and IBRgE, (IDEXX laboratories, USA) in BoHV-1 vaccinated bulls/herds. Serum ELISA results were classified as positive or negative following kit-manufacturer positive cut-off values. BoHV-1 analyses were completed by commercial laboratories; BoHV-1 lysate by National Milk Laboratories Ltd. (NML UKAS) (UK), and BoHV-1 gE by Enfer Diagnostics Ltd. (Ireland, ISO 1509001/2000).

### Descriptive data on the use of stock bulls on Irish dairy farms

#### Herds

Herds were classified according to a previous study [16] in different categories in relation to the calving season (spring-calving *vs.* non-spring-calving), number of bulls per farm (no bull *vs.* 1 bull *vs.* >1 bull), region (high density dairy *vs.* low density dairy), herd size (31–65 cows *vs.* 66–99 cows *vs.* >99 cows), type of farming enterprise (dairy livestock only *vs.* mixed livestock (farms with other cattle enterprises)), herd vaccination status (vaccinated *vs.* unvaccinated). Farms were also classified with regard to the origin of the bulls on each farm i.e. all bulls homebred *vs.* at least 1 bull purchased.

With regard to disease status, two herd classification methods were employed. Firstly herds were classified as seropositive or seronegative for each of the diseases BVD or BoHV-1 according bulk milk ELISA results [16]. For the purpose of this study, seasonal trends in bulk milk ELISA readings were not taken into account. Instead the average of all four ELISA readings was used for herd classification.

Secondly, herds were classified on the basis of having ‘evidence of recent viral circulation’ i.e. those herds having at least one weanling serologically positive for either BVDV or BoHV-1. Herds that did not record a positive weanling were classified as ‘not having evidence of recent viral circulation’ [16].

Finally, herds were classified according to ‘overall bull BoHV-1 status’, positive herds having at least one serologically positive bull in the farm.

#### Stock bulls

A questionnaire survey was used to determine the IBR vaccination status of each study farm, the vaccine product used, and the date the vaccine was administered. In the case of purchased bulls, the IBR vaccination history prior to purchase was not available. Any purchased bull testing positive for IBRgB, therefore, was re-tested using the IBRgE assay, as only marked vaccines (gE deleted) are available in republic of Ireland, in order to rule out prior vaccination as the source of seropositivity.

Bulls were categorised on the basis of breed and herd of origin (homebred *vs.* purchased). As a number of homebred bulls had moved off and back to the herd of origin, bulls were also classified according to their number of off-farm movements prior the time of sampling. Other categories included vasectomized status (vasectomized [Vas] *vs.* not vasectomized [NVas]), BoHV-1 individual vaccination status (vaccinated [Vacc] *vs.* unvaccinated [Unvacc]), and region in which the study herd was located (Region 2: high density dairy *vs.* Region 1: low density dairy). With regard to disease status, all individual animals were classified as positive or negative for BVD virus or BoHV-1 antibody.

#### Movement data of stock bulls

The national identification ear-tag number of each bull sampled was recorded during the farm visit. The availability of this unique and fully traceable animal identifier allowed specific demographic information on each bull to be sourced through the ICBF database. In addition, as all cattle movements in Ireland require a movement permit which is recorded to a central database, the number of between farm movements that each bull made prior to sampling were studied retrospectively.

#### Data analysis

Descriptive analyses were completed in Excel (MS Office 2010). Pearson’s chi-squared, Fisher’s exact, univariable and multivariable logistic regression, were carried out using Stata (Version 11).

Number of bulls per farm, number of vasectomized bulls, number of bulls per farm across regions, and number of non-homebred bulls was investigated. The age of each bull on the date of sampling was calculated, as well as the number of total movements recorded per bull and the time spent in the study herd prior to sampling.

#### Factors associated with BVD and BoHV1 status of stock bulls

Examination of relationships between BoHV-1 seropositivity of individual bulls and a number of independent variables was completed. Independent variables investigated comprised the age of the bulls at the sampling time, movements between farms pre-sampling, herd of origin (purchased *vs.* homebred) and reproductive use of the bulls (vasectomized *vs.* no vasectomized). Only BoHV-1 seropositivity of individual bulls was investigated using logistic regression due to the small number of BVD virus-positive bulls.

#### Factors associated with dairy herd status for BVD and BoHV1

Prior the construction of the final regression model an univariable analysis was completed. Those variables recording *P* values of ≤0.15 in univariable analyses were included in multivariable models. A manual backwards elimination with a forward step was used to build models with variables recording *P*-values of ≤0.05 maintained. Second level interactions deemed biologically significant were also included.

Logistic regression was used to examine the relationships of BVD herd status, BoHV-1 herd status, and evidence of recent viral circulation (categorical variables), as the dependent variables by a number of independent variables. Independent variables included having a bull, number of bulls, herd of origin of bulls and having vasectomized bulls in the farm. To investigate the relationships between the purchase of bulls and the herd status only herds with purchased bulls that had spent at least 3 months in the farm prior the sampling were considered.

## Results

### Descriptive data

#### Herds

A total of 305 study herds took part in the study and 529 individual breeding bulls were sampled. Of the 305 participating farms, 235 (77 %) had at least one bull involved in the breeding process, and 70 (23 %) had no bulls in the farm. Of the 235, 68 (29 %) farms had bulls which were homebred only. The remaining 167 (71 %) herds contained at least one purchased bull. The proportion of herds in each bull classification is outlined in Table 1 according to the number of bulls per farm across the region, herd size, herd of origin of bulls, type of enterprise, presence of vasectomized bulls in the farm and vaccine status of the herd.

**Table 1:** Descriptive data of herds that took part in the study. Study herds are classified in relation to region, origin of stock bulls, enterprise, vasectomized bulls in the farm, calving season, herd size and vaccination status. The proportion of herds within bull categories (no bull, 1 bull, >1 bull) across herd classifications is shown below

Herd classification	No bull	1 bull	>1 bull
(*n*)	% (*n*)	% (*n*)	% (*n*)
Overall proportion of herds	23 % (70)	36 % (109)	41 % (126)
Region			
Region 1 (99)	29 % (29)	33 % (33)	37 % (37)
Region 2 (206)	20 % (41)	37 % (76)	43 % (89)
Herds with all bulls were homebred			
All bulls homebred (68)		35 % (24)	65 % (44)
Purchased bulls (167)		51 % (85)	49 % (82)
Enterprise			
Dairy only (140)	25 % (35)	34 % (48)	41 % (57)
Mixed livestock species (164)	21 % (35)	37 % (60)	42 % (69)
Herds with vasectomized bulls			
Vasectomized bulls (41)		20 % (8)	81 % (33)
No Vasectomized (194)		52 % (101)	48 % (93)
Calving season			
Spring (265)	21 % (56)	35 % (92)	44 % (117)
Not spring (40)	35 % (14)	42 % (17)	23 % (9)
Size score			
31–65 cows (81)	26 % (21)	48 % (39)	26 % (21)
66–99 cows (98)	27 % (26)	39 % (38)	35 % (34)
> 99 cows (126)	18 % (23)	25 % (32)	56 % (71)
BVD Vaccine			
Yes (187)	22 % (41)	35 % (65)	43 % (81)
No (118)	25 % (29)	37 % (44)	38 % (45)
IBR Vaccine			
Yes (36)	22 % (8)	22 % (8)	56 % (20)
No (269)	23 % (62)	38 % (101)	39 % (106)

#### Stock bulls

In relation to the individual bull data 243 out of the 529 bulls analysed for this study were non-homebred. The number of bulls of each breed and vasectomized bulls in each breed is shown in Table 2. In total, 474 out of 529 bulls were used for actual breeding, with a further 55 vasectomised bulls involved in the process of heat detection. Of the 474 bulls not vasectomized 242 (51 %) were purchased, and the remainder were homebred. In contrast, only one of the vasectomized bulls was purchased, 54 (98 %) of them being home born. The average age of bulls in the study was 2.3 years (range 8 months to 10.5 years). The distribution of the individual bulls according to the herd of origin, region, vaccine status, and BVD and BoHV-1 individual status is shown in Table 3. The average of time spent by purchased bulls in the study herd was 595 days (range 1 to 3453 days). Only 14 bulls belonging to 9 different herds had spent less than 3 months in the study herd prior to the sampling point. Of these herds, five had purchased other bulls more than 3 months prior to sampling.

**Table 2:** Descriptive data of stock bulls showing the proportion of different breeds and vasectomized bulls in each breed

Breed	No vasectomized bulls	Vasectomized bulls
Simental	4	0
Rotbunt	2	0
Belgian Blue	2	0
Limousin	11	0
Hereford	50	0
Charolais	7	0
Aberdeen Angus	105	3
Holstein Friesian	129	6
Jersey	15	0
Saler	1	0
Norweigan Red	12	0
Friesian	42	0
Jersey Cross	77	1
Unknown	17	45
Total	474	55

**Table 3:** Descriptive data about the individual stock bulls according to the herd of origin (homebred or purchased), region, vasectomy, vaccine status, BVD virus status and BoHV-1 serological status

	Number of participating bulls
Herd of origin	
Homebred	286
Purchased	243
Vasectomy	
Vasectomized	55
No Vasectomized	474
Region	
Low dairy density (1)	172
High dairy density (2)	354
Vaccine IBR	
Yes	454
No	75
BVD virus status	
Positive	2
Negative	527
IBR sero status	
Positive	87
Negative	434

#### Movement data of stock bulls

**Fig. 1:**
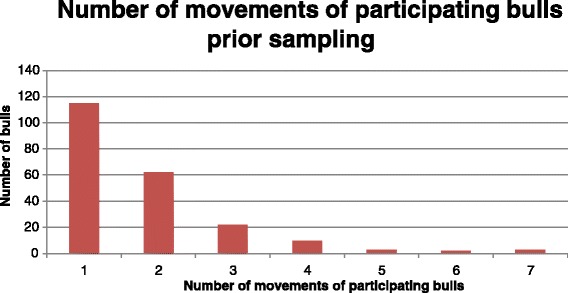
Descriptive data recording the number of stock bulls recording movements prior to sampling point

Movement data relating to 486 bulls, of the 529 total participants, was available for the period prior to sampling. Demographic information on the 43 remaining animals was not available and was, therefore, not included in the statistical analysis. Examination of movement data highlighted that 217 bulls had recorded at least 1 between farm movement, and 40 bulls recorded more than three movements prior the sampling point. The average number of movements was 1.8 (range 1 to 7). The number of animals recording between-farm movement prior the sampling is included in Fig. 1.

#### BVD and BoHV1 status of stock bulls

In all, the BoHV-1 status of 8 bulls couldn’t be confirmed and these animals were excluded from statistical analysis. Two bulls out of 529 tested positive for BVD virus yielding an apparent prevalence of 0.4 %. One bull, aged 9 months at the time of sampling, was homebred; the other aged 21 months was purchased. The apparent prevalence of BoHV-1 amongst bulls tested was 16.7 % (*n* = 87). Regarding the use of vaccines, only 75 bulls were vaccinated against BoHV-1 on the farm on which they residing at the time of the study. A total of 16 (21.3 %) tested seropositive for BoHV-1, using the gE assay. Also, 12 purchased bulls positive for BoHV-1 (test gB) were classified as negative after being re-tested using gE.

With regard to herd demography, the prevalence of bull exposure to BoHV-1 in region 1 (low dairy- density) was 18 %, meanwhile in region 2 (high dairy-density) 20 % of the bulls were detected positive. No significant differences were found between regions (OR = 1.17, 95 % CI = 0.74, 1.86, *P* = 0.49).

#### Factors associated with BVD and BoHV1 status of stock bulls

Logistic regression highlighted significant differences in BoHV-1 exposure between purchased and homebred bulls, with purchased bulls three times more likely than homebred bulls to be categorised as positive for BoHV-1 (Table 4). Increasing age was also found to be a risk factor for BoHV-1 seropositivity.

**Table 4:** Factors associated BoHV1 status of stock bulls, using logistic regression analysis. Independent variables including age of individual bulls, movements pre-sampling, herd of origin, and vasectomy

Dependent variable	Independent variable	Odds ratio	Confidence interval (95 %)		*P* value
BoHV-1 classification	Age of the bulls				
POSITIVE *vs.* NEGATIVE	2 years *vs.* 1 year	5.15	1.89	14.03	0.001
	3 years *vs.* 1 year	12.78	4.46	36.61	0
	4 years or older *vs.* 1 year	28.94	9.35	89.5	0
	Movement pre-sampling *vs.* no movement	1.32	1.04	1.67	0.019
	Purchased *vs.* Homebred	3.08	1.51	6.29	0.002
	Non vasectomized *vs.* vasectomized^a^	3.16	0.89	11.20	0.074

^**a**^Included for the purposes of highlighting a trend

The number of movements pre-trial was significantly associated with an exposure to BoHV-1, with animals with one or more recorded movements being 1.3 times more likely to be seropositive (*P* = 0.019). Also, the bulls with higher numbers of movements prior to sampling were more likely to be seropositive (Table 5). Furthermore, a tendency amongst non-vasectomized bulls was observed, being three times more likely to be seropositive (OR = 3.16, 95 % CI = 0.89, 11.2, *P* = 0.07).

**Table 5:** Logistic regression results of relationship between BoHV-1 stock bull status as dependent variable and number of movements between farms of the individual bulls as independent variable

Dependent variable	Independent variable	Odds ratio	Confidence interval (95 %)		*P* value
IBR classification	1 movement *vs.* 0 movement	1.18	0.64	2.18	0.59
POSITIVE *vs.* NEGATIVE	2 movements *vs.* 0 movement	3.27	1.74	6.16	0
	3 movements *vs.* 0 movement	2.88	1.03	8.05	0.04
	4 movements or more *vs.* 0 movement	6.24	2.32	16.75	0

#### Factors associated with dairy herd status for BVD and BoHV1

Results of the final multivariable logistic regression model of herd status are shown in Table 6. Significant associations were found between the BVD herd status and the presence of purchased bulls in the farm, with farms that purchased bulls three times more likely to be bulk milk seropositive for BVD. A tendency was noticed for farms which kept vasectomized bulls to be almost 4 times more likely to test bulk milk seropositive for BVD than farms without vasectomized bulls (OR 3.77, 95 % CI = 0.93, 15.31, *P* = 0.064).

**Table 6:** Factors associated with dairy herd status for BVD and BoHV1 using logistic regression analysis. Dependent variables included BVD herd status, BoHV-1 herd status, and evidence of recent viral circulation

Dependent variable	Independent variable	Odds ratio	Confidence interval (95 %)		*P* value
BVD herd status	Purchased bulls in the herd *vs.* all homebred	3.45	1.2	9.9	0.02
POSITIVE *vs.* NEGATIVE	Region 2 *vs.* Region 1	4.61	1.74	12.17	0.002
	66–99 cows *vs.* 31–65 cows	4.06	1.15	14.34	0.029
	Vaccinated *vs.* no vaccinated	8.78	3.13	24.62	0
	Recent circulation viral *vs.* no recent circulation	16.55	3.31	82.68	0.001
	Vasectomized bulls in the herd *vs.* no vasectomized bulls^a^	3.77	0.93	15.31	0.064
IBR herd status	>1 bull *vs.* 1 bull	2.13	1.08	4.19	0.027
POSITIVE *vs.* NEGATIVE	Region 1 *vs.* Region 2^b^	1.86	0.96	3.62	0.064
IBR recent circulation viral	Region 1 *vs.* Region 2	2.8	1.11	7.01	0.028
Yes *vs.* No	>99 cows *vs.* 31–65 cows	6.71	1.44	31.03	0.015
	Purchased bulls in the herd *vs.* all homebred	3.9	1.07	14.22	0.039
IBR overall Bull status	>1 bull *vs.* 1 bull^c^	1.83	0.95	3.54	0.068
Positive *vs.* negative	Purchased bulls in the herd *vs.* all homebred	2.73	1.28	5.82	0.009
	>99 cows *vs.* 31–65 cows	1.80	1.19	2.75	0.005

^**a,b,c**^Included for the purposes of highlighting a trend

Regarding BoHV-1 herd status, a significant association with the number of bulls was recorded. Farms with more than one bull were twice as likely to be categorised as BoHV-1 positive over those who had a single bull (*P* = 0.027). Similarly to BVD herd status, farms with purchased bulls were approximately four times more likely to be categorised as having evidence of recent BoHV-1 circulation (*P* = 0.039). Although significant associations weren’t found between the presence of purchased bulls and BoHV-1 herd status (bulk milk and heifer serology), farms with purchased bulls were almost three times more likely to have at least one positive bull on the farm over farms where all the bulls were home born (*P* = 0.009).

## Discussion

BVD and BoHV-1 are contagious viral diseases of cattle and highly prevalent in Ireland (16). Although there are a number of studies carried out in beef and dairy cattle, it would appear that no studies have been carried out to investigate the degree of infection in the stock bull population. Despite the economic advantages of artificial insemination (AI) over natural service (NS), a high number of dairy farmers in Ireland that still rely on NS, compared to other European countries [18]. Among the participating farms in the current study, 77 % had at least one bull involved in the breeding process, whereas in Northern Europe, Israel or Japan, 80 to 90 % of dairy farmers use AI almost exclusively for breeding cattle [18].

A number of countries have reported BVD PI prevalence of between 0.4 and 1.1 %. The prevalence of PIs amongst Irish calves ear-notched as part of the national BVD eradication scheme in 2013 was 0.68 %^2^, which has fallen to 0.46 % in 2014 and 0.32 % in 2015. It was not that surprising therefore, that two bulls out of 529 tested were positive for BVD virus in the current study. On reporting of initial BVDv results, the two study farmers involved elected to cull the virus positive animals without re-test to confirm PI status. The presence of clinical signs consistent with mucosal disease was identified in one bull (a purchased bull), the history of the second (a homebred ill-thrifty calf with recurring clinical issues), the low Ct values on PCR, and the absence of BVD antibodies, would suggest that both were BVD PIs [19]. PI animals are the main source of circulating BVDv leading to rapid secondary transmission once introduced to a herd. Bulls are also likely to be in very close contact with cows during the first trimester of pregnancy, and therefore may have a disproportionate impact on the generation of new PI animals the following year [20]. It is important in countries not yet engaging in BVD eradication, therefore, to monitor the breeding bull population as a potential source of BVDv.

It is also of note that one of the BVDv positive bulls identified was vasectomized. This bull was homebred, underweight and ill-thrifty as a calf, and could therefore not be sold at an early age with herd cohorts. The calf was therefore maintained on the farm and subsequently vasectomized, highlighting the ease with which a PI can be maintained on farm and may ultimately contribute to the breeding process on a dairy farm. Based on these results, the use of vasectomized bulls and choice of animal to be vasectomized deserves careful consideration on dairy farms. Vasectomized bulls act as a cost-saving measure for heat detection purposes [21], but if the animal is not chosen carefully, the long term economic consequences can easily outweigh any benefit. The importance of ensuring the absence of PI animals on a farm cannot be overstated.

Based on the results of the current study, the likelihood of introducing of BoHV-1 seropositive bull would appear greater than BVDv positive bulls. While a previous Irish study reported 6 % of beef bulls selected for performance testing by the national cattle breeding centre as being seropositive for BoHV-1 [22], just over 16 % of dairy bulls in the current study were positive. No difference between dairy and beef herds in terms of BoHV-1 was reported previously [23]. The difference in bull prevalence recorded may reflect differences in age groups investigated as a much older cohort of animals were included in the current study. A number of studies have reported older animals to be more likely to be seropositive for BoHV-1 [9, 24, 25], which is in agreement with the results obtained in this study.

Purchased bulls were three times more likely to be BoHV-1 seropositive in the current study. Cattle trade and movement, in general, have been reported by a number of authors as major risk factors for the spread of BoHV-1 [9, 26, 27]. The number of movements recorded for individual bulls in this study was remarkable and an unsurprising trend was highlighted whereby the more ‘between-farm’ movements recorded by a particular bull, the higher the likelihood of being BoHV-1 seropositive. This may be due to the higher number of risk interactions (shows, markets, transport) that these bulls experience. This aligns with results from previous surveys [27] which recorded a higher predisposition of BoHV-1 seropositive herds to buy cattle and participate in shows. These results highlight the importance of seeking a full diagnostic and movement history prior to purchasing a breeding bull. These findings should be considered in other jurisdictions where stock bulls remain an important part of the breeding programme.

An additional noteworthy finding of this study was the significant association between purchase of bulls with both BVD herd status and BoHV-1 viral circulation. The high probability of having recent BoHV-1 viral circulation in herds that purchased bulls has also been identified in previous studies [9], where purchase of cattle (as opposed to just stock bulls) identified as a major direct risk factor for BoHV-1 infection. It was not possible in the current study to obtain records of additional purchased cattle on study farms as individual identifiers for other cattle were not available. This is a potential weakness of the study, as purchase of a bull may be a proxy for purchase of additional high risk cattle onto a farm. It has been reported previously that bulls are more likely to be seropositive than cows [9], making them a higher risk purchase and further investigation is required to more clearly highlight the contribution of stock bulls to disease introduction on dairy farms. Nevertheless, this study has highlighted a clear association between purchase of bulls and an undesirable BVD and BoHV-1 status.

Farms with non-homebred bulls were three times more likely to be bulk milk positive for BVD than those farms where all the bulls were homebred, in contrast to BoHV-1, where no relationship was found. This most likely relates to the differences in the dynamic of spread of both viruses across a herd population. In the case of BoHV-1, a large proportion of latent carriers exist within an adult dairy herd allowing for the possibility of continuing re-infection from within the herd itself. In contrast to this, transmission of BVDv in the adult herd is dependent on contact with a PI. Many PIs die, however, before reaching breeding age [28], leading to a break in re-infection. The culling rate of dairy cows in Ireland (25 %) [29], coupled to death of PIs at a young age, may lead to dairy herds having more cyclical BVDv transmission rather than the continuous re-infection possible in unvaccinated BoHV-1 infected dairy herds. The contribution of a purchased infected bull, therefore, in increasing the bulk milk antibody levels in a herd, is most likely greater for BVDv. Regardless of the differences in associations detected between BVDv and BoHV-1, these results again support the hypothesis that introducing purchased bulls in the farm could led to an increased risk for both viral infections.

Two factors were significantly associated with an increased BoHV-1 herd serostatus. This included farm location and the number of bulls present on each farm. Farms in Region 1 (lower cattle density) had a higher probability of being positive for BoHV-1, which has already been investigated by other authors [16], who propose that it could be due to the higher proportion of beef cattle in the region and a reduced probability of implementing biosecurity measures. The association between number of stock bulls and BoHV-1 herd seropositivity is a novel finding. Considering that bulls are more at risk of being seropositive than cows [9] and that once the virus is introduced in the herd the infection is quickly disseminated amongst herd mates [30]; the higher number of bulls maintained in the farm could lead to a higher risk of BoHV-1 infection. Again this highlights the potential role of stock bulls in viral disease dissemination on farm making them a cohort of animals that should undergo disease screening prior to movement or purchase.

## Conclusion

Minimal attention has been dedicated internationally to the role of stock bulls in transmission of infectious diseases in dairy farms. This study recorded widespread use of stock bulls on Irish dairy farms, a high degree of ‘between-farm’ movement of these animals, and the potential for bulls to act as a vector of disease between farms. Further studies into the role of stock bulls in transmission of viral diseases, in countries where NS is common practice, is required, as is the role of these breeding animals in transmission of other infectious pathogens.

## Footnotes

1 http://www.cso.ie/

2 http://www.animalhealthireland.ie/news.php?id=74
